# Supplementary figures and images for: Research on the prediction of English topic richness in the context of multimedia data
Source: PeerJ Comput Sci. 2024 Apr 16;10:e1967. doi: 10.7717/peerj-cs.1967 (PMC11042032; doi:10.7717/peerj-cs.1967)

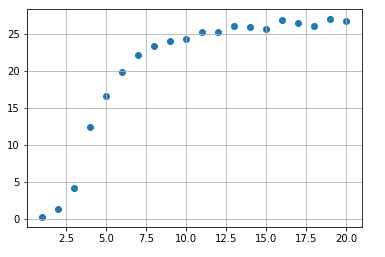

Supplement: Supplemental Information 1 [file peerj-cs-10-1967-s001.zip › code/fig/bleu.png]

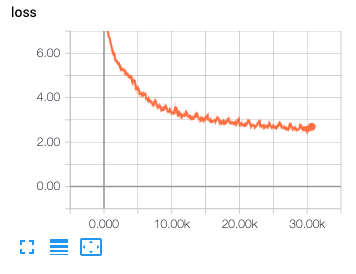

Supplement: Supplemental Information 1 [file peerj-cs-10-1967-s001.zip › code/fig/loss.png]

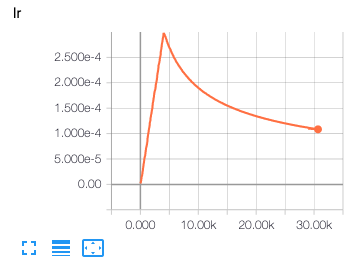

Supplement: Supplemental Information 1 [file peerj-cs-10-1967-s001.zip › code/fig/lr.png]

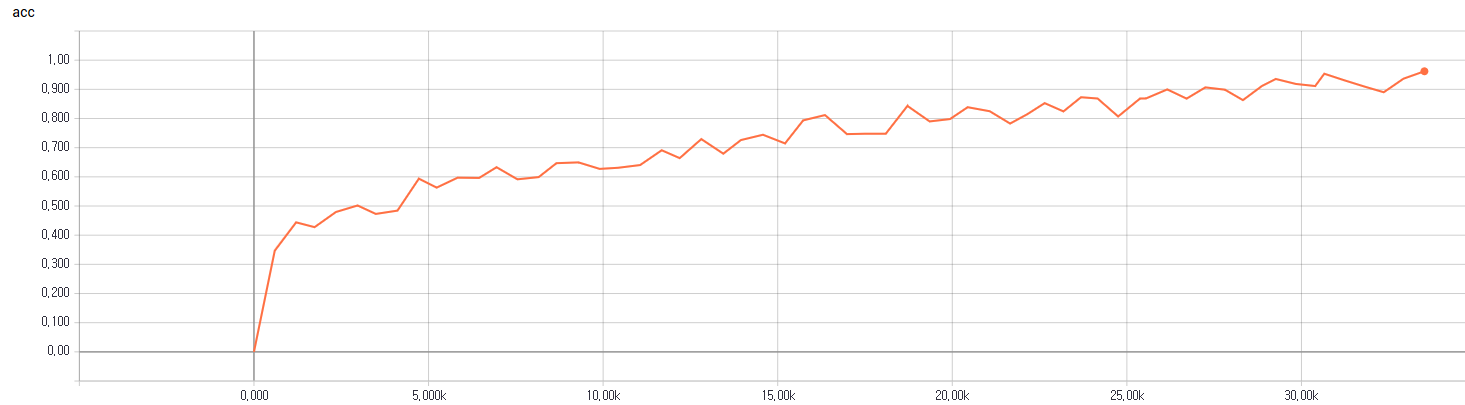

Supplement: Supplemental Information 1 [file peerj-cs-10-1967-s001.zip › code/tf1.2_legacy/fig/accuracy.png]

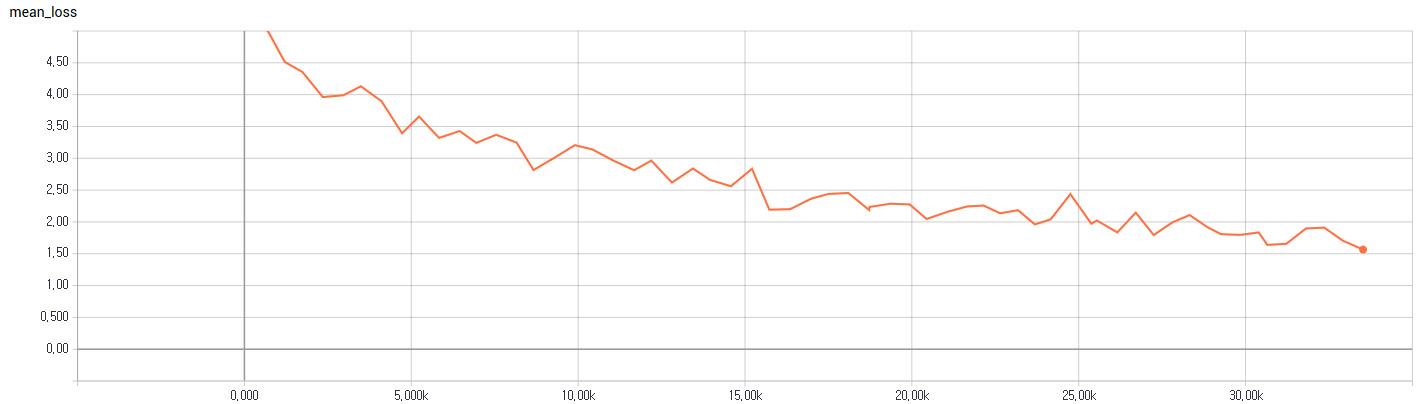

Supplement: Supplemental Information 1 [file peerj-cs-10-1967-s001.zip › code/tf1.2_legacy/fig/mean_loss.png]
